# Sensitivity and specificity of the video head impulse test in the diagnosis of vestibular migraine – A systematic review and meta-analysis

**DOI:** 10.1016/j.bjorl.2026.101806

**Published:** 2026-04-02

**Authors:** Francisco Campelo da Fonseca Neto, Raquel Mezzalira, Vinicius Domene, Carlos Takahiro Chone

**Affiliations:** Universidade Estadual de Campinas (UNICAMP), Departamento de Otorrinolaringologia e Cirurgia de Cabeça e Pescoço, Campinas, SP, Brazil

**Keywords:** Vestibular migraine, Video head impulse test, Vestibulo-ocular reflex

## Abstract

**Objective:**

To estimate the proportion of patients with VM who have abnormal vHIT results and to determine the sensitivity and specificity of the test to detect VM.

**Methods:**

This study followed PRISMA guidelines to conduct a meta-analysis on the accuracy of vHIT in VM patients. A literature search was performed in ten indexes databases (PubMed, PubMed PMC, Embase, Scopus, Web of Science, Cochrane Library, BVS, ProQuest, Epistemonikos and MEDLINE) without time restrictions, using the descriptors “Vestibular Migraine” AND “Video Head Impulse Test”. Studies were selected based on the PICO strategy (Patient, Intervention, Comparison, and Outcome). Statistical analysis used the random-effects method to pool results, assessing heterogeneity among studies with Cochran’s*Q* test and the *I*^2^ statistic. The accuracy of vHIT was estimated through sensitivity, specificity, and predictive values. A significance level was 5%. This review was not registered in PROSPERO (International Prospective Register of Systematic Reviews) because it evaluates previously published observational studies of diagnostic performance without patient-level data collection.

**Results:**

Nine studies published between 2016 and 2023 were included in the meta-analysis. The prevalence of abnormal results was 43%. vHIT had a sensitivity of 42%, specificity of 89%, positive predictive value of 83%, and negative predictive value of 54% for VM diagnosis. Saccades was observed in 36% of cases. Normal VOR gain was found in 22% of cases, while low VOR gain was observed in 12%. The gains in the lateral, anterior, and posterior semicircular canals ranged from 0.87 to 0.90. Except for normal VOR gain, all other outcomes showed high heterogeneity across studies.

**Conclusion:**

43% of VM patients exhibited abnormalities in vHIT. The test demonstrated low sensitivity and high specificity to detect VM.

**Level of evidence:**

1.

## Highlights

• vHIT showed 42% sensitivity and 89% specificity for vestibular migraine.
• Abnormal vHIT findings in 43% of patients with vestibular migraine.
• Covert or overt saccades were reported in 36% of the included cases.
• High specificity suggests vHIT may help rule in vestibular migraine.

## Introduction

Vestibular Migraine (VM) is characterized by recurrent dizziness and vertigo, associated with typical migraine symptoms.^1^ The diagnosis is based on specific criteria proposed by Neuhauser in 2001 and revised in 2012 and 2022 by the Bárány Society and the International Headache Society.^2^^,^^3^ It is a common cause of spontaneous acute vertigo lasting from minutes to days, as well as recurrent positional vertigo that can mimic benign paroxysmal positional vertigo.^4^

The exact mechanism by which VM affects vestibular function remains unclear. Trigeminovascular system activation is one of the theories proposed to explain the pathophysiological link between migraine and dizziness. Trigeminal activation may trigger vestibular symptoms during a migraine attack through reciprocal connections with the vestibular nuclei in the brainstem.^5^

Vestibular tests have provided useful tools for diagnosing dysfunctions in different vestibular pathways, contributing to the understanding of VM pathophysiology. Among the available tests for vestibular function assessment, the Video Head Impulse Test (vHIT) provides an objective evaluation of all semicircular canals and is a physiological and easily applicable test.^6^ This is particularly relevant in the assessment of migraine patients, as they exhibit a high degree of intolerance to sensory stimuli. The test uses high-frequency head movements in different directions while their eye movements are recorded using a high-speed camera. This allows for the assessment of ocular reflexes and the detection of any vestibular asymmetry. Additionally, vHIT can be used to monitor patient progression over time and assess treatment effectiveness.^7^

Some studies have shown that patients with VM often exhibit abnormalities in the Vestibulo-Ocular Reflex (VOR) in the vHIT, such as compensatory eye movements known as saccades and reduced VOR gain. These alterations suggest vestibular dysfunction, which may be related to the disease’s pathophysiology. Furthermore, these findings have been associated with a higher frequency and severity of vestibular symptoms.^8^

However, there is still no standardized pattern of vHIT results in VM patients. Studies present a wide range of findings, sometimes with conflicting results.

This study aimed to analyze, through a systematic review and meta-analysis, the proportion of patients with VM who have abnormal vHIT results and to determine the sensitivity and specificity of the test to detect VM.

## Methods

### Search strategy and data sources

This study followed the recommendations of the Preferred Reporting Items for Systematic Reviews and Meta-Analyses (PRISMA) method.^1^ The literature search was conducted without restrictions on the publication period in the following indexed databases: BVS, Cochrane Library, Embase, Epistemonikos, ProQuest, PubMed, PubMed PMC, Scopus, Web of Science and MEDLINE. Studies published until March 2024 were considered. A combination of controlled vocabulary (EMTREE) and free terms was used as the search strategy for this study. The Boolean search expression applied was: (“vestibular migraine” OR “migraine related vestibulopathy” OR “migraine-associated vertigo” OR “migraine-related vertigo” OR “migrainous vertigo”) AND (“Video Head Impulse Test” OR “video-head impulse test” OR “vHIT” OR “video head impulse testing”). The reference lists of the included studies were also manually searched to identify additional eligible articles. Duplicate records were removed using Rayyan (Qatar Computing Research Institute).

### Eligibility criteria for study selection

The studies selected for this meta-analysis were established using the PICO strategy (Patient, Intervention, Comparison, and Outcome), with the target population consisting of individuals with vestibular migraine aged 18-years or older diagnosed according to the diagnostic criteria of the Bárány Society 2012 and 2022, which include recurrent vestibular symptoms of moderate or severe intensity lasting between 5 min and 72 h, a current or previous history of migraine, migraine features during at least 50% of vestibular episodes, and exclusion of other diagnoses.^2^^,^^3^ The intervention considered was the performance of the video Head Impulse Test (vHIT). Thus, eligible studies for this research had a consistent analysis of this test in patients diagnosed with vestibular migraine. The studies had to describe the stimulus used for vHIT (including the number of head impulses, amplitude in degrees, and peak velocity in degrees per second) and outline the criteria used to determine vHIT abnormalities (including gains in lateral and/or vertical semicircular canals and the presence or absence of refixation saccades).

The exclusion criteria were as follows: (1) Inappropriate target population (age under 18-years), (2) In vitro experimental studies, (3) Inappropriate study types such as simple reviews, abstracts, letters to the editor, and case reports, (4) Insufficient clinical information, (5) Studies published in languages other than Portuguese, English, or Spanish, (6) Improper outcome and (7) Studies in which the diagnostic criteria for vestibular migraine were not clearly defined.

**Fig. 1:**
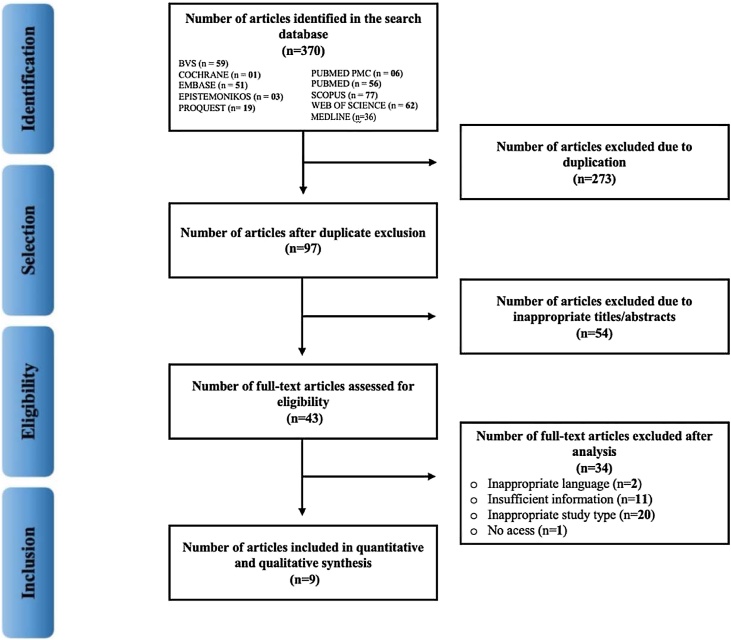
Flowchart of article selection.

Initially, the titles and abstracts of all articles were independently reviewed by two researchers. According to the eligibility criteria, the preselected articles were then read in full for inclusion in the meta-analysis. In cases of disagreement during the selection process, a third reviewer performed an independent analysis using Rayyan to compare and resolve conflicts during study selection. The screening process flow diagram is presented in Fig. 1.

### Data extraction

Data extraction was performed in a standardized way, including the year of publication, authors, sample size, mean age of the included patients, study design, and the proportion of vestibular migraine patients with abnormal vHIT results.

### Methodological quality assessment

The Agency for Health Care Research and Quality (AHRQ) checklist was used to assess methodological quality of the included studies. This checklist consists of 11 criteria for evaluating studies included in research, namely: source of information, inclusion and exclusion criteria, time period, consecutive patients, blinding, quality assurance, explanation of exclusions, control of confounding factors, handling of incomplete data, complete data collection, and patient follow-up. Each criterion is scored as 1 if present in the study or 0 if absent. Thus, high methodological quality corresponds to articles with a score of 8 or higher. Scores between 5 and 7 were classified as moderate quality (moderate risk of bias), and scores of 4 or less as low quality (high risk of bias), according to AHRQ criteria.^10^ A sensitivity analysis excluding low-quality studies was not necessary because all included studies were classified as high methodological quality (Table 1).

**Table 1:** Quality control of the selected studies according to the Agency for Healthcare Research and Quality (AHRQ) criteria.

Article	A	B	C	D	E	F	G	H	I	J	K	Total
Koç et al., 2022	1	1	1	1	1	0	0	1	1	1	1	9
Calic et al., 2020	1	1	1	1	0	0	0	1	1	1	1	8
Elsherif et al., 2018	1	1	1	1	0	0	1	1	1	1	0	8
Fu et al., 2021	1	1	1	1	0	0	1	1	1	1	0	8
Waissbluth et al., 2023	1	1	1	1	0	0	1	1	1	1	0	8
Kang et al., 2016	1	1	1	1	0	0	1	1	1	1	0	8
Young et al., 2017	1	1	1	1	0	0	1	1	1	1	0	8
Elsherif et al., 2020	1	1	1	1	0	0	1	1	1	1	0	8
Du et al., 2022	1	1	1	1	0	0	1	1	1	1	0	8

A, Information source; B, Inclusion or exclusion criteria; C, Time period; D, Consecutive patients; E, Blinding; F, Quality assurance; G, Explanation of exclusions; H, Control of confounding factors; I, Removal of incomplete data; J, Data integrity; K, Patient follow-up; 1, Information present; 0, Information absent or uncertain.

Table 2, Table 3, Table 4 summarize the characteristics of the studies and the parameters assessed in the vHIT. Table 5 describes the data used to calculate the sensitivity and specificity of vHIT in the diagnosis of vestibular migraine.

**Table 2:** Evaluated characteristics of the selected studies.

Article	Year	Type of study	Patients (M/F)	Mean Age	Abnormal VHIT
Koç et al.	2022	Case-control	84 (12/72)	40.0	44 (52.3%)
Calic et al.	2020	Retrospective cohort	10 (5/5)	47.4	10 (100%)
ElSherif et al.	2018	Case-control	80 (11/69)	39.1	21 (26.3%)
Fu et al.	2021	Retrospective cohort	41 (15/26)	49.3	13 (32%)
Waissbluth et al.	2023	Retrospective cohort	60 (11/49)	45.0	26 (43.3%)
Kang et al.	2016	Retrospective cohort	81 (19/62)	50.8	9 (11%)
Young et al.	2017	Case-control	89ª	48.0	5 (5.6%)
ElSherif et al.	2020	Retrospective cohort	25 (3/22)	41.7	9 (36%)
Du et al.	2022	Retrospective cross-sectional	14 (3/11)	44.2	12 (86%)

M, Male; F, Female; vHIT, Video Head Impulse Test.

ª The study does not provide a sex-based division.

**Table 3:** Parameters used for performing vHIT in the analyzed studies.

Article	Year	Stimulus Used	Alteration Criteria
Koç et al.	2022	> 20 head impulses | A 10–15 ° | D 150–200 ms | VP 150–200 °/s	VOR gain < 0.8 (lateral canals) | VOR gain < 0.7 (vertical canals) | Saccades
Calic et al.	2020	> 20 head impulses	VOR gain < 0.86 (lateral canals) | VOR gain < 0.65 (anterior canals) | VOR gain < 0.68 (posterior canals) | Saccades
ElSherif et al.	2018	> 20 head impulses | A 10–15 ° | VP 150 °/s	VOR gain < 0.8 (lateral canals) | VOR gain < 0.7 (vertical canals) | Saccades
Fu et al.	2021	> 20 head impulses | A 10–20 ° | VP 150–200 °/s	VOR gain < 0.8 (lateral canals) | VOR gain < 0.63 (vertical canals) | Saccades
Waissbluth et al.	2023	> 20 head impulses | VP 150–300 °/s	VOR gain < 0.8 (lateral canals) | VOR gain < 0.7 (vertical canals) | Saccades
Kang et al.	2016	A 5–15 ° | VP 150–250 °/s	VOR gain < 0.8 (lateral canals) | AG ≥ 8% | Saccades
Young et al.	2017	> 20 head impulses	VOR gain < 0.8 | Saccades
ElSherif et al.	2020	> 20 head impulses | A 10–20 ° | VP 100–200 °/s	VOR gain < 0.8 (lateral canals) | VOR gain < 0.7 (vertical canals) | Saccades
Du et al.	2022	> 10 head impulses | VP >150 °/s	VOR gain < 0.8 | Saccades

A, Amplitude, D, Duration; VP, Peak Velocity; AG, Gain Asymmetry; VOR, Vestibulo-Ocular Reflex; vHIT, Video Head Impulse Test.

**Table 4:** Characterization of saccades and mean VOR gains.

Articles	Year	Saccades with normal VOR gain	Saccades with low VOR gain	Absence of saccades	Mean VOR gains (Lateral canals ± DP)	Mean VOR gains (Anterior canals ± DP)	Mean VOR gains (Posterior canals ± DP)
Koç et al.	2022	24 (28.5%)	20 (23.8%)	40 (47.6%)	‒	‒	‒
Calic et al.	2020	‒	‒	‒	0.61 (± 0.20)	0.63 (± 0.20)	0.70 (± 0.20)
ElSherif et al.	2018	15 (18.8%)	6 (7.5%)	59 (73.8%)	0.87 (± 0.23)	1.15 (± 0.30)	1.09 (± 0.28)
Fu et al.	2021	‒	‒	‒	‒	‒	‒
Waissbluth et al.	2023	11 (18.3%)	6 (11.7%)	43 (71.6%)	0.97 (± 0.12)	0.88 (± 0.10)	0.82 (± 0.11)
Kang et al.	2016	‒	‒	‒	‒	‒	‒
Young et al.	2017	‒	‒	‒	0.95 (± 0.12)	0.90 (± 0.19)	0.85 (± 0.16)
ElSherif et al.	2020	7 (28%)	2 (8%)	16 (64%)	‒	‒	‒
Du et al.	2022	‒	‒	‒	0.92 (± 0.07)	0.93 (± 0.06)	0.95 (± 0.05)

VOR, Vestibulo-Ocular Reflex; DP, Standard Deviation.

**Table 5:** Calculation of sensitivity and specificity of vHIT to detect Vestibular Migraine.

Article	N	TP	FP	FN	TN	Sensibility	Specificity	PPV	NPV
						(95% CI)	(95% CI)	(95% CI)	(95% CI)
Du et al. 2022	28	12	4	2	10	0.86	0.71	0.75	0.83
						(0.57, 0.98)	(0.42, 0.92)	(0.48, 0.93)	(0.52, 0.98)
Koç et al. 2022	162	44	8	40	70	0.52	0.90	0.85	0.64
						(0.41, 0.63)	(0.81, 0.95)	(0.72, 0.93)	(0.54, 0.73)
Elsherif et al. 2020	45	9	2	16	18	0.36	0.90	0.82	0.53
						(0.18, 0.57)	(0.68, 0.99)	(0.48, 0.98)	(0.35, 0.70)
Elsherif et al. 2018	120	21	3	59	37	0.26	0.92	0.88	0.39
						(0.17, 0.37)	(0.80, 0.98)	(0.68, 0.97)	(0.29, 0.49)
4 artigos	355	86	17	117	135	0.42	0.89	0.83	0.54
						(0.35, 0.49)	(0.83, 0.93)	(0.75, 0.90)	(0.47, 0.60)

N, Total number of subjects; TP, True Positive; FP, False Positive; FN, False Negative; TN, True Negative; PPV, Positive Predictive Value; NPV, Negative Predictive Value.

### Statistical analysis

Statistical analyses were performed using *R* software (version 4.3.0; The R Foundation for Statistical Computing, 2023). Meta-analyses were conducted using random-effects models estimated by the Restricted Maximum Likelihood (REML) method, which was selected to account for between-study variability and provide unbiased estimates of heterogeneity. For proportion data, a logit transformation was applied to stabilize variances, and pooled estimates were backtransformed for interpretation. Confidence intervals were calculated using the Wald approximation under the Normal distribution. Analyses were performed using the metafor package, which enables robust estimation of effect sizes under REML.

Heterogeneity across studies was evaluated using Cochran’s *Q* test, and its magnitude was quantified using the *I*^2^ statistic, which represents the percentage of total variability attributable to heterogeneity rather than sampling error. Values of *I*^2^ greater than 50% were considered indicative of substantial heterogeneity.

The accuracy of vHIT in the diagnosis of vestibular migraine was estimated by calculating the sensitivity, specificity, positive predictive value, and negative predictive value of the test. The level of significance adopted was 5% (p < 0.05).

## Results

Nine articles published between 2016 and 2023 were selected for the meta-analysis after applying the eligibility criteria described in the Methods section. The sample size of the studies ranged from 10 to 89. Due to the small number of analyzed articles, it was not possible to assess publication bias. The rate of VM patients that exhibited abnormalities in vHIT was 43%.

Table 6 presents a summary of the meta-analysis for each investigated outcome. High heterogeneity among the studies was observed, as evidenced by both Cochran’s *Q* test and the *I*^2^ measure in all analyzed outcomes [*I*^2^ > 50%], except for the estimated proportion of normal VOR gain.

**Table 6:** Estimation of outcomes by meta-analysis.

Outcomes	Number of articles	Number of subjects	Effect	CI (95%)	I^2^	Q Test
Abnormal VHIT	9	484	43%	22%‒64%	97%	p < 0.01
Absence of saccades	4	249	64%	52%‒77%	80%	p < 0.01
Normal VOR gain	4	249	22%	17%‒28%	9%	p = 0.35
Low VOR gain	4	249	12%	5%‒19%	68%	p = 0.02
Lateral Canal	5	253	0.87	0.76‒0.99	90%	p < 0.01
Anterior Canal	5	253	0.90	0.75‒1.06	95%	p < 0.01
Posterior Canal	5	253	0.89	0.76‒1.01	96%	p < 0.01

CI, Confidence Interval; *I*^2^, Heterogeneity quantification; VOR, Vestibulo-Ocular Reflex; VHIT, Video Head Impulse Test.

**Fig. 2:**
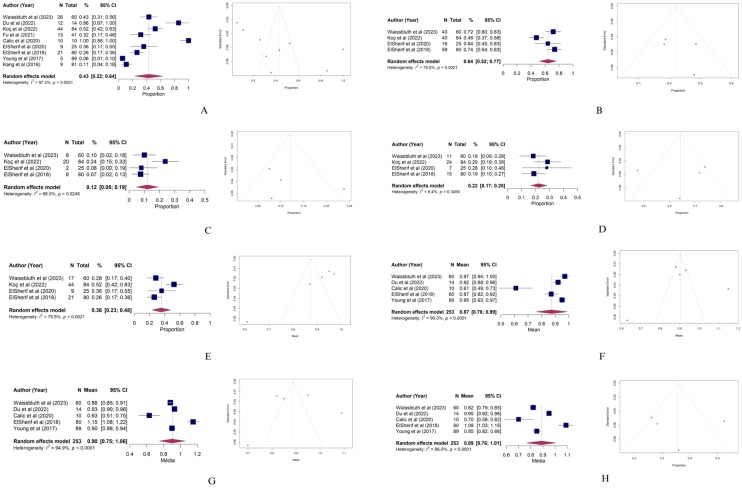
Forest plots and funnel plots of the outcomes. (A) Forest plot and funnel plot of Altered vHIT; (B) Forest plot and funnel plot of Absence of Saccades; (C) Forest plot and funnel plot of Normal VOR Gain; (D) Forest plot and funnel plot of Low VOR Gain; (E) Forest plot and funnel plot of Presence of Saccades; (F) Forest plot and funnel plot of Lateral Semicircular Canal Gain; (G) Forest plot and funnel plot of Anterior Semicircular Canal Gain; (H) Forest plot and funnel plot of Posterior Semicircular Canal Gain.

Fig. 2 display the forest plots and funnel plots for each analyzed outcome. All outcomes were statistically significant; however, high heterogeneity was observed among the studies, except for the estimated proportion of normal VOR gain. The assessment of publication bias using a funnel plot is considered statistically valid when a meta-analysis includes ten or more studies. This is because, with a smaller number of publications, sampling variability is high, and any asymmetry in the plot may occur merely by chance. Therefore, the funnel plots presented in this study are purely exploratory and informative.

The accuracy of vHIT in diagnosing vestibular migraine can be seen in Table 5. The test showed low sensitivity, high specificity and high positive predictive value.

## Discussion

The pathophysiology of Vestibular Migraine (VM) remains unclear, but there appear to be multiple factors leading to abnormalities in the central nervous system and potassium homeostasis in the inner ear.^11^ Some authors suggest the presence of a genetic factor in migraine and some genes such as CACNA1A, NOTCH3, TREX1, and COL4A1 are involved. These genes would be related to ion channel abnormalities, leading to potassium accumulation in the extracellular space and the cortical spreading depression. This phenomenon is associated with the release of substance P and Calcitonin Gene-Related Peptide (CGRP) by trigeminal fibers, resulting in increased vascular permeability and subsequent local inflammatory response. Thus, acute episodes of hearing loss and/or vertigo associated with migraine can be explained by vasospasm of the cochlear and/or vestibular branches of the internal auditory artery.^11^

Therefore, neurovascular inflammation due to activation of the trigeminovascular system, implicated in the pathophysiology of migraine, likely involves both central vestibular pathways and the inner ear. This could explain the different alterations found in tests evaluating vestibular function. The results of this systematic review and meta-analysis showed that approximately 43% of patients with VM had abnormalities of the VOR detected by vHIT. This finding suggests that VM significantly impacts vestibular function, resulting in changes in oculomotor reflexes mediated by the semicircular canals. When compared to similar studies, other reviews also indicate a frequent association between VM and vestibular dysfunction, although the patterns of alteration vary considerably.^6^ This variation may be attributed not only to the recurrent nature of VM, which means that the lesion is transient, but mainly because VM is a condition in which varied clinical manifestations and different degrees of vestibular system impairment are involved.^12^ However, the high heterogeneity among the studies included (*I*^2^ = 97%) reflects methodological inconsistencies and limitations in controlling population variables. One example is the disparity observed in the criteria for defining abnormalities in vHIT, the application of the tests, and patient selection, as described by Waissbluth et al. 2023.^13^

All alterations analyzed in our review showed significance, but the inconsistency among the studies limits the generalization of the findings and highlights the need for standardization in research protocols.

When evaluating the absence of refixation saccades, it was observed that 64% of patients with VM did not show refixation saccades. Factors such as age, symptom duration, and methodology applied may influence these results.^8^ The absence of saccades should be interpreted as a complementary data point within a broader diagnostic approach.

On the other hand, the presence of corrective saccades was observed in 36% of patients, reflecting more pronounced vestibular dysfunction in a considerable subgroup. Recent studies indicate that larger amplitude saccades may be related to the severity of vestibular dysfunction, emphasizing the relevance of this feature in the clinical context.^14^ When associated with low VOR gain, suggests vestibular deficits.^15^ In contrast, small amplitude and scattered saccades suggest that some vestibular dysfunction persists even in the absence of symptoms.^16^ Some authors suggest that refixation saccades are more important and reliable in interpreting vHIT than VOR gain.^8^^,^^15^

Covert saccades are relatively common and can be present even with normal VOR gain. They could be explained by a disturbed inhibitory input from the cerebellar flocculus to the vestibular nuclei consequent to the classic migraine cortical spreading depression, leading to a disturbance in the spontaneous firing of the nuclei.^8^

The main vestibular modulation systems are the commissural pathways, cross-inhibitory system, and the vestibular-cerebellar connections. The first has an important rebalancing function between the nuclei following an input deficit, and the second is GABAergic and inhibitory system, controlling vestibular reflexes and visual-vestibular calibration.^17^^,^^18^ Individuals with migraine have subclinical cerebellar dysfunction, and it is possible to hypothesize the influence of these limitations on vestibular pathways.^19^ Thus, in a cerebello-vestibular pathway dysfunction, the reduction in the inhibition of the vestibular system by the GABAergic pathway justifies the hyperactivity to stimuli observed in the vHIT.^20^ In the migraine crisis, early acceleration and premature deceleration during stimulation of the semicircular canals have been reported, suggesting involvement of the medial vestibular nucleus, flocculus, and other neuro-integrator structures. This reinforces the coexistence of both peripheral and central signs during a vestibular migraine attack.^21^

The proportion of patients with normal VOR gain was 22%, with low heterogeneity (*I*^2^ = 9%), indicating greater consistency among the studies. This result suggests that the VOR reflex arc is intact, and that vestibular dysfunction is a central feature of VM, but it also points to the existence of different phenotypic subgroups. Exploring such differences may contribute to a better understanding of the pathophysiology of VM.^7^ Low VOR gain was found in 12% of patients, representing a smaller proportion compared to other alterations identified in vHIT. The release of CGRP in trigeminal endings may explain the association between migraine and vestibular symptoms. CGRP-positive neurons are in the ampulla of the semicircular canals, as well as in the utricular and saccular maculae.^22^ Cellular ionic homeostasis is mediated by canalicular function. An ionic defect in the canal due to a genetic mutation may lead to depolarization of the hair cell and consequently a vertiginous attack.^23^ Therefore, it is possible that VM is related to endolymphatic hydrops, which justifies the vestibular hypofunction with low VOR gain observed in some patients.

The moderate heterogeneity among the studies (*I*^2^ = 68%) may be attributed to differences in symptom severity and VM duration among the patients included in the studies. This observation highlights the need to stratify patients based on these variables in future studies.^5^ The high VOR gain in patients with migraine, without vertigo or imbalance, has also been described, likely due to impairment of the GABAergic cerebellar pathway over the vestibular nuclei, culminating in vestibular system hyperactivity.^20^ In the studies included in this meta-analysis, there were no reports of high VOR gain. The average VOR gains in the lateral, anterior, and posterior semicircular canals were similar, ranging from 0.87 to 0.90. These values suggest that vestibular dysfunction in VM affects all three canals in a uniform manner, but once again, the high heterogeneity (*I*^2^ between 90% and 96%) suggests that the studies used divergent criteria to measure and interpret the gains. Studies such as Moher et al. (2009) highlight the importance of standardizing protocols to improve the comparability of findings.^9^

The results of the studies analyzed in this review allow us to suppose that vestibular dysfunction is an intrinsic feature of patients with VM, regardless of whether they manifest symptoms. There is cerebello-vestibular pathways dysfunction that reduce the physiological inhibition of the vestibular system by the cerebellar efferents, resulting in its hyperactivity.^20^ The results of the vHIT accuracy to detect VM reveal important information. The average sensitivity of 42% indicates that the test has low capacity to identify all VM cases, limiting its use as a screening tool on its own. This limitation is likely related to the variability in symptom expression and differences in alteration patterns detected across the studies.^15^

The specificity of 89% demonstrates that vHIT is a reliable tool in the differential diagnosis of patients suspected of having VM. This means that the test has good accuracy in differentiating VM from other vestibular conditions, such as vestibular neuritis and benign paroxysmal positional vertigo, reducing the possibility of false positives. This diagnostic capability is essential to ensure that patients receive targeted and appropriate treatments for their specific conditions, avoiding unnecessary interventions.^24^ The Positive Predictive Value (PPV) of 83% confirms its utility for diagnoses in selected populations. This data suggests that a positive result on vHIT in these patients indicates a high probability that VM is the underlying condition. This aspect makes vHIT particularly valuable in clinical settings where VM is highly suspected, allowing greater confidence in the diagnosis and contributing to therapeutic decision-making. On the other hand, the Negative Predictive Value (NPV) of 54% reinforces that vHIT should not be used alone as an exclusion tool. This indicates that a negative result on the test does not reliably exclude the possibility of VM, especially in populations with a high prevalence of the condition. In other words, although the vHIT shows limited sensitivity (42%), its high specificity (89%) indicates that abnormal findings are strongly suggestive of true vestibular dysfunction, supporting its use in selected clinical scenarios. Unlike caloric testing, which assesses low-frequency vestibular responses, the vHIT evaluates the Vestibulo-Ocular Reflex (VOR) at high frequencies and may detect abnormalities even when caloric results are normal.^17^ The vHIT is also useful in differentiating Vestibular Migraine (VM) from unilateral vestibulopathy, where VOR gain reduction is more commonly observed.^20^ Compared to VEMP, which assesses otolithic function, the vHIT provides complementary information regarding semicircular canal function,^14^^,^^21^ supporting its role as an adjunctive tool in the diagnostic assessment of VM. These tests are best utilized in a complementary fashion, contributing to a more comprehensive and accurate diagnostic approach. Furthermore, given the sensory hypersensitivity commonly observed in VM, especially during acute episodes, the vHIT offers a less invasive and more tolerable alternative for functional vestibular evaluation. Therefore, integrating vHIT with other diagnostic tests and clinical data is essential to improve diagnostic accuracy and ensure a comprehensive evaluation.

The data underscores that vHIT has significant limitations as an isolated diagnostic tool for VM, especially due to the high heterogeneity of results and the clinical variability of the condition and we recognize these factors as an important limitation of the meta-analysis. Standardizing protocols, both in the application and interpretation of results, is crucial to improve the reliability and reproducibility of the test.

Another limitation of the present meta-analysis is that several of the included studies had relatively small sample sizes, which may reduce the statistical power to detect differences and limit the generalizability of the pooled estimates. Small samples increase the risk of unstable effect size estimates and wide confidence intervals, potentially influencing the accuracy of meta-analytic results. Therefore, the findings of this review should be interpreted cautiously and validated in future studies with larger and more representative populations.^25^

However, although it is not an ideal tool, vHIT is useful as a complementary test to detect VM, particularly due to its good specificity and positive predictive value.

## Conclusion

In the current study, 43% of the VM patients exhibited abnormalities in vHIT. The sensitivity of the test to detect VM was 42% and the specificity was 89%. These findings indicate that although vHIT has low sensitivity, it demonstrates high specificity, which supports its usefulness in ruling in VM when results are abnormal. However, due to the high heterogeneity among the included studies and methodological variability, the strength of the evidence should be considered moderate. Therefore, vHIT should not be used as a standalone diagnostic tool for VM, but rather as a complementary test integrated with clinical evaluation and other vestibular assessments. Future studies with standardized protocols and larger samples are necessary to clarify the diagnostic value of vHIT in vestibular migraine.

## Declaration of Generative AI and AI-assisted technologies in the writing process

During the preparation of this manuscript, the authors utilized Open AI Chat GPT to enhance the quality of writing and ensure that the language is more comprehensive and appropriately aligned with scientific understanding. After using this tool, the authors reviewed and edited the content as needed and took full responsibility for the content of the publication.

## Funding

There is no financial or material supports.

## Data availability statement

The authors declare that all data are available in repository.

## Declaration of competing interest

The authors declare no conflicts of interest.

## References

[1] Salmito M.C., Ganança F.F. (2021). Video head impulse test in vestibular migraine. Braz J Otorhinolaryngol..

[2] Lempert Thomas (2022). Vestibular migraine: diagnostic criteria (update). J Vestibular Res.

[3] Lempert Thomas (2012). Vestibular migraine: diagnostic criteria. J Vestibular Res.

[4] Von Brevern Michael (2004). Migrainous vertigo presenting as episodic positional vertigo. Neurology.

[5] Shin Jung H. (2014). Altered brain metabolism in vestibular migraine: comparison of interictal and ictal findings. Cephalalgia.

[6] Calic Zeljka (2020). Vestibular migraine presenting with acute peripheral vestibulopathy: clinical, oculographic and vestibular test profiles. Cephalalgia Reports.

[7] Kang Woo Seok (2016). Vestibular function tests for vestibular migraine: clinical implication of video head impulse and caloric tests. Front Neurol.

[8] Elsherif Mayada (2018). Video head impulse test (vHIT) in migraine dizziness. Journal of otology.

[9] Moher David (2009). Preferred reporting items for systematic reviews and meta-analyses: the PRISMA statement. Ann Intern Med.

[10] Guise J.M. (2017). AHRQ series on complex intervention systematic reviews – paper 7: PRISMA-CI elaboration and explanation. J Clin Epidemiol.

[11] Baloh Robert W. (2020).

[12] Blödow Alexander (2014). Caloric stimulation and video-head impulse testing in Ménière’s disease and vestibular migraine. Acta Otolaryngol (Stockh).

[13] Waissbluth Sofia (2023). Vestibular and Oculomotor Findings in Vestibular Migraine Patients. Audiology Research.

[14] Young Allison S. (2021). Clinical, oculographic, and vestibular test characteristics of vestibular migraine. Cephalalgia.

[15] Koç Ahmet, Akkiliç Elvan Cevizci (2022). Evaluation of video head impulse test during vertiginous attack in vestibular migraine. Acta Otorhinolaryngologica Italica.

[16] Du Yi (2022). Analysis of video head impulse test saccades data in patients with vestibular migraine or probable vestibular migraine. J Otol.

[17] De Zeeuw C.I., Berrebi A.S. (1995). Postsynaptic targets of Purkinje cell terminals in the cerebellar and vestibular nuclei of the rat. Eur J Neurosci.

[18] Li Peng (2019). Purkinje cells of vestibulocerebellum play an important role in acute vestibular migraine. J Integr Neurosci.

[19] Harno Hanna (2003). Subclinical vestibulocerebellar dysfunction in migraine with and without aura. Neurology.

[20] Bernetti Laura (2018). Subclinical vestibular dysfunction in migraineurs without vertigo: a clinical study. Acta Neurol Scand.

[21] Na Seunghee (2019). Video head impulse findings in the ictal period of vestibular migraine. J Neurol.

[22] Seo Toru (2022). Endolymphatic hydrops presumption tests for patients with vestibular migraine. Acta Otolaryngol (Stockh).

[23] Casani Augusto P. (2009). Otoneurologic dysfunctions in migraine patients with or without vertigo. Otol Neurotol.

[24] Elsherif Mayada (2020). Eye movements and imaging in vestibular migraine. Acta Otorrinolaringol Esp.

[25] Valentine Jeffrey C., Pigott Therese D., Rothstein Hannah R. (2010). How many studies do you need? A primer on statistical power for meta-analysis. J Educ Behav Stat.

